# *CDKN2A* and *CDK4* mutation analysis in Italian melanoma-prone families: functional characterization of a novel CDKN2A germ line mutation

**DOI:** 10.1054/bjoc.2001.1991

**Published:** 2001-09

**Authors:** G Della Torre, B Pasini, S Frigerio, R Donghi, D Rovini, D Delia, G Peters, T J G Huot, G Bianchi-Scarra, F Lantieri, M Rodolfo, G Parmiani, M A Pierotti

**Affiliations:** Department of Experimental Oncology, Istituto Nazionale Tumori, Via Venezian, Milan, 1 20133, Italy; Medical Genetics Unit, Istituto Nazionale Tumori, Via Venezian, Milan, 1 20133, Italy; Department of General Surgery “C”, Istituto Nazionale Tumori, Via Venezian, Milan, 1 20133, Italy; Department of Prevention, A.S.L. of Lecco, 22053, Italy; Imperial Cancer Research Fund Laboratories, London, United Kingdom; Institute of Biology and Genetics, University of Genova, Genova, 16100, Italy

**Keywords:** familial melanoma, *CDKN2A* and *CDK4* genes, germ line mutations

## Abstract

Physical interaction between CDKN2A/p16 and CDK4 proteins regulates the cell cycle progression through the G1 phase and dysfunction of these proteins by gene mutation is implicated in genetic predisposition to melanoma. We analysed 15 Italian melanoma families for germ line mutations in the coding region of the *CDKN2A* gene and exon 2 of the *CDK4* gene. One novel disease-associated mutation (P48T), 3 known pathological mutations (R24P, G101W and N71S) and 2 common polymorphisms (A148T and Nt500 G>C) were identified in the *CDKN2A* gene. In a family harbouring the R24P mutation, an intronic variant (IVS1, +37 G>C) of uncertain significance was detected in a non-carrier melanoma case. The overall incidence of *CDKN2A* mutations was 33.3%, but this percentage was higher in families with 3 or more melanoma cases (50%) than in those with only 2 affected relatives (25%). Noteworthy, functional analysis established that the novel mutated protein, while being impaired in cell growth and inhibition assays, retains some in vitro binding to *CDK4/6*. No variant in the p16-binding region of *CDK4* was identified in our families. Our results, obtained in a heterogeneous group of families, support the view that inactivating mutations of *CDKN2A* contribute to melanoma susceptibility more than activating mutations of *CDK4* and that other genetic factors must be responsible for melanoma clustering in a high proportion of families. In addition, they indicate the need for a combination of functional assays to determine the pathogenetic nature of new *CDKN2A* mutations. http://www.bjcancer.com © 2001 Cancer Research Campaignhttp://www.bjcancer.com


*British Journal of Cancer* (2001) **85**(6), 836–844
© 2001 Cancer Research Campaign
doi: 10.1054/ bjoc.2001.1991, available online at http://www.idealibrary.com on IDEAL
http://www.bjcancer.com

### *CDKN2A* and *CDK4* mutation analysis in Italian melanoma-prone families: functional characterization of a novel CDKN2A germ line mutation

**G Della Torre[1], B Pasini[2], S Frigerio[1], R Donghi[4], D Rovini[3], D Delia[1], G Peters[5], TJG Huot[5], G Bianchi-Scarra[6], F Lantieri[6], M Rodolfo[1], G Parmiani[1] and MA Pierotti[1]**

[1]Istituto Nazionale Tumori – Department of Experimental Oncology, Via Venezian, 1 20133 Milan, Italy; [2]Istituto Nazionale Tumori – Medical Genetics Unit, Via Venezian, 1 20133 Milan, Italy; [3]Istituto Nazionale Tumori – Department of General Surgery "C", Via Venezian, 1 20133 Milan, Italy; [4]Department of Prevention, A.S.L. of Lecco, 22053, Italy, [5]Imperial Cancer Research Fund Laboratories, London, United Kingdom; [6]Institute of Biology and Genetics, University of Genova, Genova 16100, Italy

**Summary** Physical interaction between CDKN2A/p16 and CDK4 proteins regulates the cell cycle progression through the G1 phase and dysfunction of these proteins by gene mutation is implicated in genetic predisposition to melanoma. We analysed 15 Italian melanoma families for germ line mutations in the coding region of the *CDKN2A* gene and exon 2 of the *CDK4* gene. One novel disease-associated mutation (P48T), 3 known pathological mutations (R24P, G101W and N71S) and 2 common polymorphisms (A148T and Nt500 G>C) were identified in the *CDKN2A* gene. In a family harbouring the R24P mutation, an intronic variant (IVS1, +37 G>C) of uncertain significance was detected in a non-carrier melanoma case. The overall incidence of *CDKN2A* mutations was 33.3%, but this percentage was higher in families with 3 or more melanoma cases (50%) than in those with only 2 affected relatives (25%). Noteworthy, functional analysis established that the novel mutated protein, while being impaired in cell growth and inhibition assays, retains some in vitro binding to *CDK4/6*. No variant in the p16-binding region of *CDK4* was identified in our families. Our results, obtained in a heterogeneous group of families, support the view that inactivating mutations of *CDKN2A* contribute to melanoma susceptibility more than activating mutations of *CDK4* and that other genetic factors must be responsible for melanoma clustering in a high proportion of families. In addition, they indicate the need for a combination of functional assays to determine the pathogenetic nature of new *CDKN2A* mutations. © 2001 Cancer Research Campaign http://www.bjcancer.com

**Keywords**: familial melanoma; *CDKN2A* and *CDK4* genes; germ line mutations

Approximately 8–12% of all melanoma cases occurs in patients with a family history of the disease (Greene and Fraumeni, 1979), suggesting genetic susceptibility as a predisposing factor to melanoma. Familial melanoma is considered a genetically heterogeneous disease as supported by different lines of investigation including genetic linkage analyses that have identified at least 2 loci, on chromosomes 9p21 and 1p36, cosegregating with melanoma susceptibility (Haluska and Hodi, 1998). The *CDKN2A* gene, at 9p21, encodes a product, known as p16, that functions as a negative regulator of the cell cycle progression at the G1–S checkpoint. It binds specifically and blocks the activity of 2 cyclin-dependent protein kinases, CDK4 and CDK6, which coordinately phosphorylate and functionally inactivate the product of the *RB* tumour suppressor gene (Ruas and Peters, 1998). Thus, changes in the expression of one of these proteins or mutations that impair their interaction may allow unchecked cell growth, contributing to cell transformation. While mutations in the *CDKN2A* gene normally interfere with binding of p16 to CDK4, rare mutated *CDK4* alleles function as dominant oncogenes as their encoded proteins escape p16-binding and inhibition thus retaining the ability to phosphorylate pRB (Zuo et al, 1996).

*Received 6 February 2001*
*Revised 16 May 2001*
*Accepted 8 June 2001*

*Correspondence to:* G Della Torre

A number of molecular studies on melanoma families from Australia, North America and Europe have been reported and the presence of germ line *CDKN2A* mutations has been detected with discordant frequencies (Haluska and Hodi, 1998). Because of the influence of different ethnic and environmental factors, and of high variations in family selection criteria, including 9p21 linkage and the size of the samples studied, the involvement of *CDKN2A* germ line mutations in melanoma families in the world is difficult to evaluate. Overall, the mutation detection rate in the families studied is approximately 20%. *CDKN2A* germ line alterations in familial melanoma are typically point mutations or small deletions and insertions in the coding region of the gene. Various mutations have been shown to cosegregate with melanoma risk in large pedigrees and to compromise the inhibitory function of p16 (Castellano and Parmiani, 1999), thus supporting their causative role in melanoma predisposition and tumorigenesis. However, as indicated by the divergent values of mutation frequencies, many families do not carry abnormalities in the coding region of the gene. Activating mutations of the *CDK4* gene are rare in the development of familial (Zuo et al, 1996; Soufir et al, 1998) or sporadic melanoma (Wolfel et al, 1995; Guldberg et al, 1997). To date, only 2–6% of all analysed melanoma families have been found to carry amino acid substitutions, specifically the replacement of arginine 24 with either cysteine or histidine (Wolfel et al, 1995; Brotherton et al, 1998). Another missense mutation in the p16-binding region (K22Q) has been demonstrated in a cell line derived from a sporadic melanoma (Tsao et al, 1998). Disruption of either codon 24 or codon 22 is likely to abrogate the interaction between CDK4 and p16 (Coleman et al, 1997; Brotherton et al, 1998).

To further elucidate the respective contributions of *CDKN2A* and *CDK4* in familial clustering of melanoma, we performed a mutational analysis of these genes in 15 Italian melanoma families. One novel and 3 already known *CDKN2A* mutations were identified mainly in families with multiple melanoma cases with an overall mutation detection rate of 33.3%. The protein expressed from the novel mutant allele was found to be normal in in vitro *CDK4*- and *CDK6*-binding activity, but defective in cell growth and cell-cycle inhibition. Mutations in the p16-binding region of *CDK4* were not detected in our families.

### MATERIALS AND METHODS

### Patients and family collection

14 melanoma patients with positive family history for the same malignancy and 1 patient with multiple primary melanomas were enrolled in the study. All patients were examined and treated at the National Cancer Institute of Milan. Patients and family members were invited to participate in this study based on research purposes. After informed consent, peripheral blood for mutation analysis was obtained from the proband in 9 families and from the proband and a second affected member in another 6 families. In the 5 families in which a *CDKN2A* mutation was found a total of 48 additional members were identified and sampled for analysis. All families were of Caucasian origin and living in Italy. Clinical information including histology, tumour site, age at diagnosis of melanoma was sought from medical records for all probands and affected relatives.

### Single strand conformation polymorphism (SSCP) analysis and DNA sequencing

For all probands, genomic DNA was extracted by phenol-chloroform from lymphoblastoid cell lines established by EBV immortalization of peripheral blood lymphocytes as described by Delia et al (1997). For other available family members, DNA was extracted from peripheral blood lymphocytes using the QIAamp Blood Kit (Qiagen). Polymerase chain reaction (PCR) products of exons 1–3 of *CDKN2A* gene were screened for mutations by SSCP analysis, as reported by Donghi et al (1993). SSCP analysis of *CDK4* gene was performed on exon 2 in all probands and in a second affected member additional to 6 probands, and on exons 2–8 in 4 probands. For *CDKN2A*, all exons, whether or not they showed a bandshift, were amplified by a new PCR from genomic DNA and analysed by direct automated (ABI PRISM Dye Terminator Cycle Sequencing kit, Perkin Elmer-La Roche) or manual (Amplitaq Cycle Sequencing Kit, Perkin-Elmer-La Roche) DNA sequencing. For CDK4, PCR products of exon 2 from all probands were screened for the R24C mutation by StuI restriction digestion, as well as SSCP, as described by Zuo et al (1996).

### Functional assays

The protein–protein interaction assay was performed as previously described (Parry and Peters, 1996) using radiolabelled components translated in vitro from plasmid DNAs. The wild-type or P48T mutant CDKN2A cDNA was cloned in the pRSET vector, to facilitate in vitro translation of His6-tagged protein, and CDKs cDNAs in pBS-KS vector. The CDKN2A cDNA was also inserted into the pBABEpuro retrovirus vector (Morgenstern and Land, 1990) to allow infection of primary fibroblasts. Two copies of the HA tag were incorporated at the amino terminus of the wild-type p16 construct in pBABEpuro. Retroviral infection of TIG-ER fibroblasts and analysis of cell proliferation effects were performed according to the previously described procedure (Ruas et al, 1999). For cell cycle inhibition assay, the CDKN2A cDNA was cloned into the BamHI site of pCMVneoBam expression vector (Baker et al, 1990), and transfected in human osteosarcoma cell line U2OS, negative for p16 expression, together with a plasmid encoding the CD20 cell surface marker (Koh et al, 1995). The p16 expression in cotransfected cells was evaluated by Western blotting and enhanced chemiluminescence detection, as previously described (Delia et al, 1997).

### RESULTS

### Characterization of the families

The clinical characteristics of the 15 families screened are summarized in Table 1, and Figure 1A, B shows the distribution of melanoma-affected relatives in the 5 pedigrees (2587, 2588, 2564, 2624, 2835) carrying *CDKN2A* germ line mutations.

The number of melanoma-affected relatives per family ranged from 1–9. In 6 families at least 3 relatives had melanoma, in 7 other families 2 first-degree relatives were affected, and 1 family presented with 2 second-degree melanoma relatives and 1 case of pancreatic cancer. In the remaining case, the proband developed 5 subsequent primary melanomas although his family history was negative. 4 of the 6 families with 3 or more melanoma cases included at least one member with multiple primary melanomas. The age at diagnosis of melanoma in the 15 probands ranged from 25 to 49 years and in 13 families at least one melanoma relative was diagnosed before age 50. In 5 families, 4 melanoma patients had an additional diagnosis of colon cancer, lip cancer or basal cell carcinoma and one additional melanoma proband also had multiple basal cell carcinomas. Among the non-penetrant *CDKN2A* mutation carriers belonging to families 2587 and 2588, 3 had a diagnosis of Hodgkin disease, oral squamous cell carcinoma and prostate cancer, respectively. Tumours other than melanoma and pancreatic cancer occurred in one or more members of a total of 7 families and they were heterogeneous for type and location.

### Mutational analysis

Probands from 15 melanoma families were initially screened for germ line mutations by SSCP analysis of the entire coding sequence of *CDKN2A* gene. A total of 6 distinct abnormally migrating bands were recognized among the amplified exons from 9 probands. By nucleotide sequence analysis, a variant band of exon 1 was found to be a novel codon 48 mutation. At the position 1 of the codon 48 there was a C to A transversion resulting in the substitution of proline (CCG) with threonine (ACG) (Figure 2A). The proband carrying the P48T is a woman of the family 2587 (III:15, Figure 1A) who developed 4 primary melanomas before age 56. For the other 3 variants, the nucleotide changes were a G to C transversion at codon 24 (R24P), an A to G transition at codon 71 (N71S), and a G to T transversion at codon 101 (G101W).

© 2001 Cancer Research Campaign

*2587*
*CDKN2A P48T*

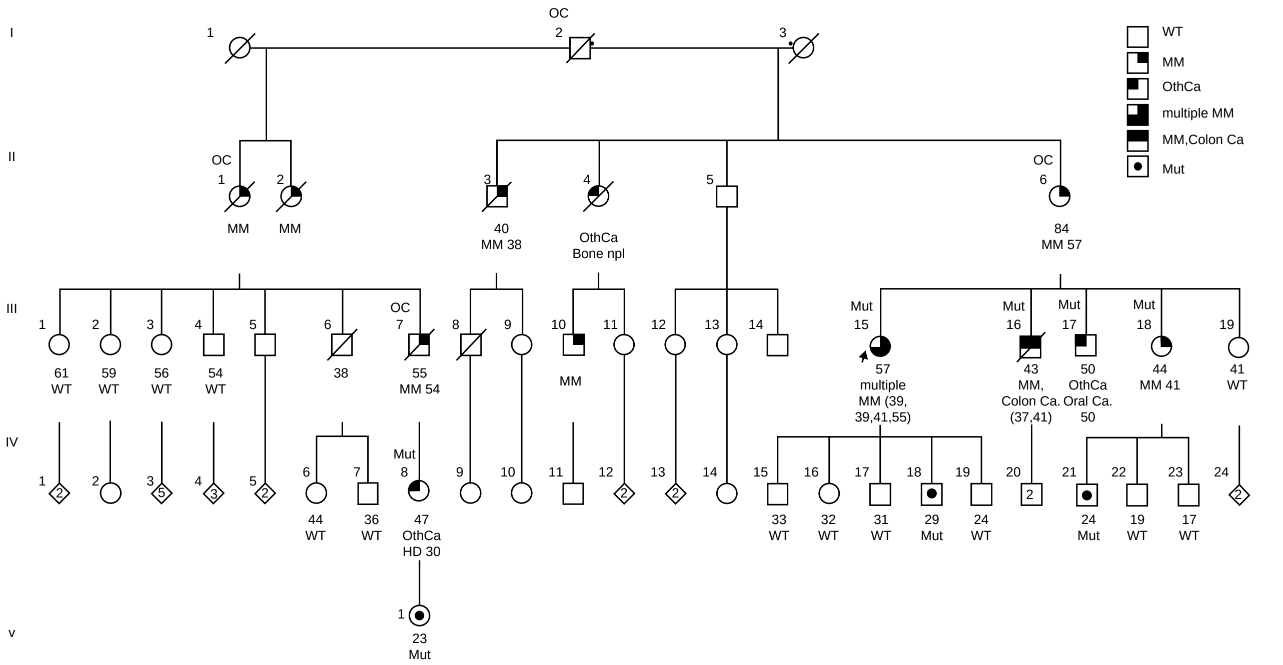

*2588*
*CDKN2A R24P*

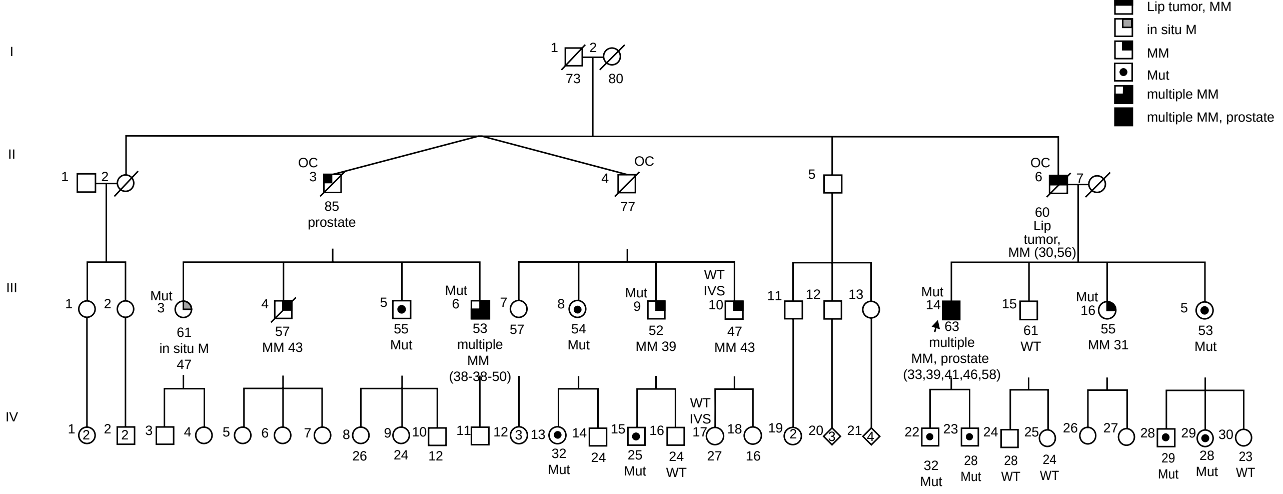

*2564*
*CDKN2A G101W*

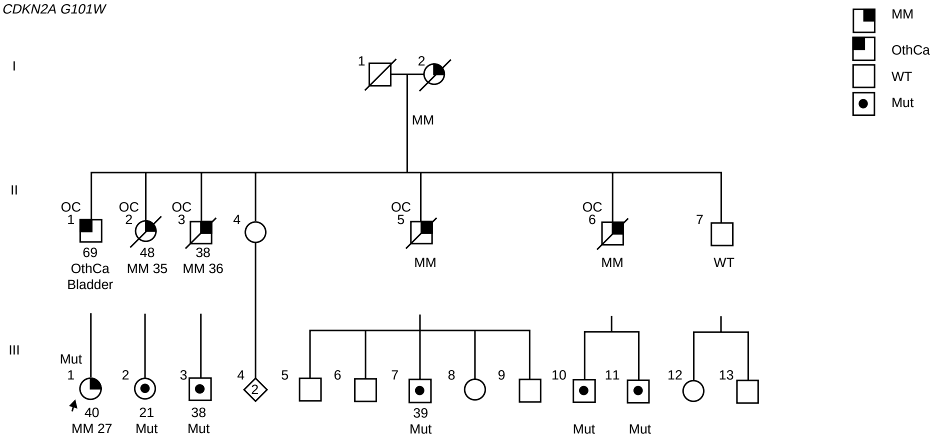

*© 2001 Cancer Research Campaign*

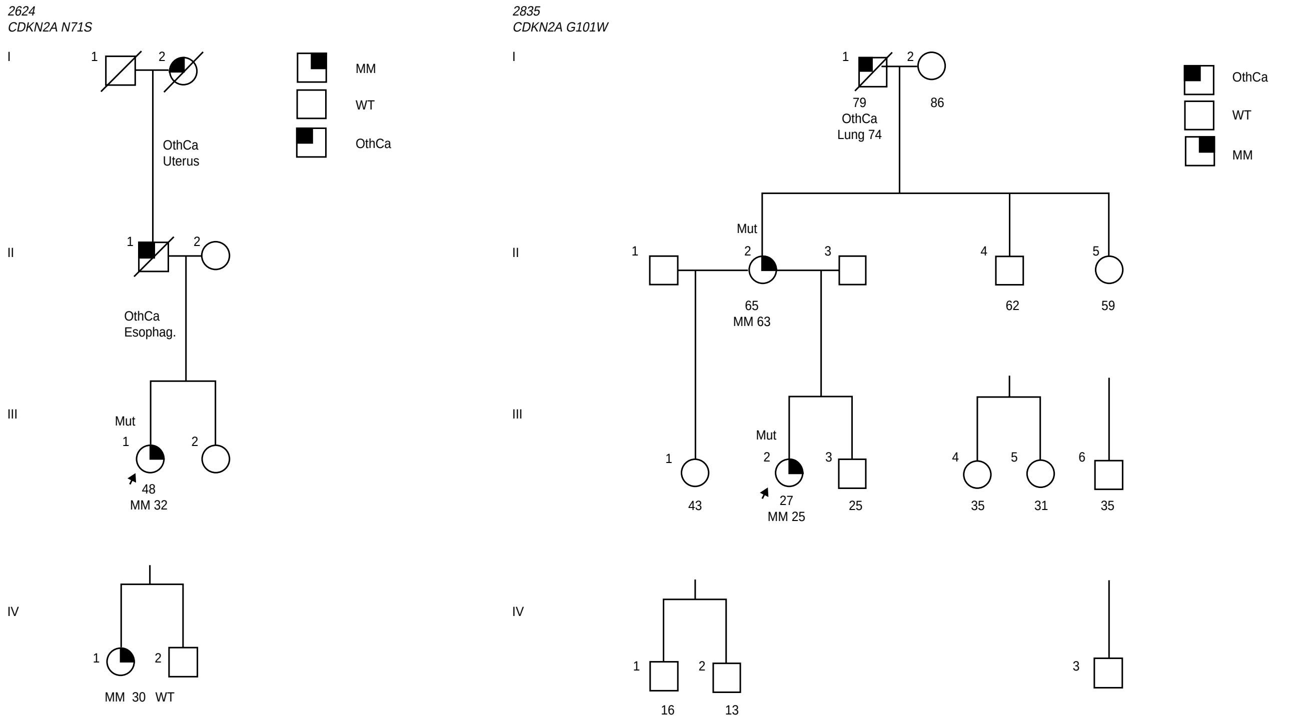

**Figure 1** Pedigrees of *CDKN2A* mutation-positive families. (**A**) Families 2587, 2588, 2564. (**B**) Families 2624, 2835. WT, non-carriers of mutation; MUT above symbols, affected mutation carriers; symbols enclosing a dot, unaffected mutation carriers; OC, obligate carrier; arrow, index case; numbers under symbols, age at present and age at diagnosis for the affected

These changes were detected in 5 of our probands (Table 1) and represent previously characterized mutations in melanoma families. The proband with the R24P mutation of family 2588 (III:14, Figure 1A) developed 5 primary melanomas (between ages 33 and 58) and later a prostate cancer. The last 2 SSCP variants were polymorphisms unrelated to melanoma risk. The G to A substitution at codon 148 of exon 2 (A148T) and the G to C substitution at Nt500 of the 3′ untranslated region (UTR) within exon 3 were detected in 2 and 6 melanoma patients, respectively, belonging to families having 1 or 2 affected relatives (Table 1). Direct DNA sequencing failed to reveal mutational changes in the 6 probands with normal SSCP migration patterns for all 3 exons investigated. In the families of 3 probands, the absence of detectable *CDKN2A* mutations was additionally confirmed on a second affected family member by SSCP analysis and direct sequencing.

Among the 3 families with extended pedigrees carrying the P48T, R24P or G101W mutations, 49 family members, including probands, were available for segregation analysis. In family 2587 (Figure 1A), direct DNA sequencing of exon 1 revealed that the P48T mutation was carried by 3/3 melanoma patients and 5/18 unaffected family members. Among the 3 affected mutation carriers one (III:18) developed a melanoma at age 41 diagnosed during the follow-up programme, and among the 5 unaffected mutation carriers one (IV:8) had Hodgkin disease when she was 30 years old, and another one (III:17) was diagnosed during follow-up, at age 50, with a well differentiated squamous cell carcinoma of the soft palate. The 5 non-penetrant carriers of the mutation ranged in age from 23 to 50 years. Out of 12 mutation carriers including obligate ones, 6 (50%) had developed melanoma. Among the 21 available members of family 2588 (Figure 1A), the R24P mutation was identified in 5/6 affected and 9/15 unaffected individuals. The age of the 9 non-penetrant carriers ranged from 25 to 55 years. Of 17 carriers and obligate carriers of the mutation, 6 (35%) had melanoma. The affected member (III:10) who did not carry the expected mutation showed heterozygosity for the polymorphism at Nt500 of the 3′ UTR and the presence of a G to C change at nucleotide +37 of intron 1 (based on GenBank accession number U12818) of unknown biological significance (Figure 2B). Both changes were also detected in his unaffected daughter (IV:17). The 3′ UTR polymorphism did not segregate with melanoma and the intron 1 variant was not encountered in any of the other 19 available family members. We have not yet been able to establish if the intron 1 variant was inherited from the paternal or the maternal lineage. Potential effects of this variant on the splicing of the gene were investigated by using the Splice View program (http://125.itba.mi.cnr.It/genebin/wwwspliceview), and no defect in RNA processing was predicted. In family 2564 (Figure 1A), the presence of the G101W mutation was investigated in 7 family members and it was identified in one case of melanoma (proband), 2/2 cases of dysplastic nevi (III:3; III:7) and 3/4 unaffected individuals. 4 affected siblings of the second generation were deceased before the beginning of the study but they represented obligate carriers. In family 2624 (Figure 1B), the N71S substitution was not identified in the unaffected son of the proband. The segregation of this variant with melanoma could not be studied, as other family members were not available for testing. In family 2835 (Figure 1B), the G101W substitution was also identified in the mother of the proband, the only available affected member of the family.

Proband and a second affected member of 5 mutation-negative families (2629, 2603, 2589, 2626, 2586) were also tested for mutations in 1200 bp of the promoter region upstream of the *CDKN2A* start codon, in the laboratory of the Center for Hereditary Tumor in Genoa by Mantelli et al (personal communication). The

*© 2001 Cancer Research Campaign*

**Table 1** Clinical and molecular characteristics of 15 Italian families

| Family members ID | No of melanoma patient/family[d] | No of melanoma patient with multiple primary melanomas | Age (years) at diagnosis of melanoma | Other tumours developed by melanoma patients or CDKN2A mutation carriers | Other tumours developed by family members | *CDKN2A* gene[c] Coding region | *CDKN2A* gene[c] Nt 500 3′ UTR | *CDK4* gene WT in exon: |
|---|---|---|---|---|---|---|---|---|
| 2587 | 9 | 1 | 39[b]–60 | colon cancer, Hodgkin disease, oral squamous cell carcinoma | 'bone' tumour | P48T | C/C | 2 to 8 |
| 2588 | 8[a] | 2 | 31–56 | prostate cancer, 'lip' tumour | – | R24P | C/C | 2 to 8 |
| 2564 | 6 | – | 27[b]–42 | – | bladder cancer | G101W | C/C | 2 to 8 |
| 2565-A | 6[a] | – | 37–62 | – | uterus cancer | wt | C/C | 2 to 8 |
| 2565-B | | | | | | wt | C/C | 2 |
| 2623 | 3 | 2 | 33[b]–35 | – | uterus cancer | wt | C/C | 2 |
| 2629-A | 3 | 1[a] | 49[b]–UNK | multiple basal cell carcinomas | – | wt | C/C | 2 |
| 2629-B | | | | | | wt | C/C | 2 |
| 2603 | 2[a](1st) | – | 21–36[b] | – | – | wt | C/C | 2 |
| 2589-A | 2(1st) | – | 31[b]–UNK | basal cell carcinoma | – | wt | C/C | 2 |
| 2589-B | | | | | | wt | C/C | 2 |
| 2624 | 2(1st) | – | <30–32[b] | – | oesophagus and uterus cancer | N71S | C/C | 2 |
| 2626-A | 2(1st) | – | 38[b]–48 | – | colon, stomach and ovarian cancer | *A148T* | *C/G* | 2 |
| 2626-B | | | | | | *A148T* | *C/G* | 2 |
| 2586-A | 2(1st) | – | 42[b]–43 | – | – | wt | *C/G* | 2 |
| 2586-B | | | | | | wt | *C/G* | 2 |
| 2764 | 2(1st) | – | 43–49[b] | basal cell carcinoma | multiple mieloma | wt | *C/G* | 2 |
| 2835-A | 2(1st) | – | 25[b]–63 | – | – | G101W | C/C | 2 |
| 2835-B | | | | | | G101W | C/C | 2 |
| 2762 | 2(2nd) | – | 49[b]–>75 | – | pancreatic cancer | wt | C/C | 2 |
| 2577 | 1 | 1 (5 primary tumours) | 37[b] | – | – | wt | *C/G* | 2 |

[a]In situ melanoma (pre-malignant melanosis) included; [b]index case; [c]Polymorphisms are shown in italics; [d]the degree of relatedness is indicated in parentheses; [UNK]Unknown age at diagnosis.

© 2001 Cancer Research Campaign

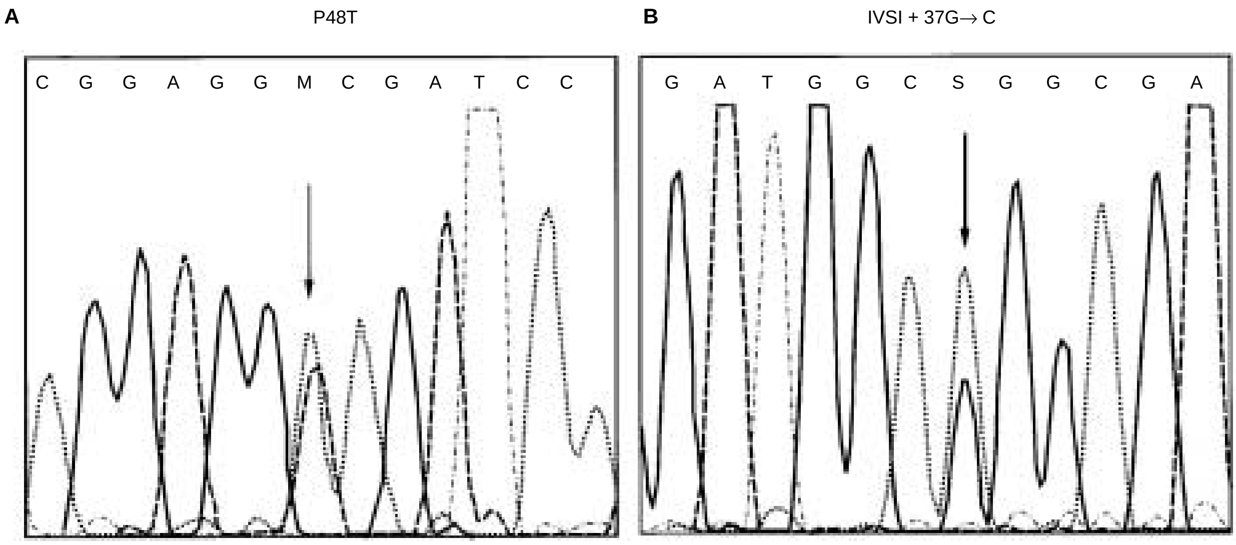

**Figure 2** *CDKN2A* gene sequence analysis. (**A**) The novel P48T germ line mutation found in the family 2587. (**B**) Identification of an *I*ntronic *V*ariant *S*equence in intron 1 (+37 G > C) in a phenocopy (III:10) of the family 2588 carrying the disease-associated R24P mutation. Arrows indicate nucleotide changes functionally deleterious –34 G > T promoter variant, the only one known to segregate with melanoma risk (Harland et al, 2000), was not detected in any of these families.

In screening the *CDK4* gene, the previously described exon 2 R24C mutation was not detected by StuI restriction digestion in any of the 15 probands analysed. SSCP analysis revealed no abnormal migration patterns either in exon 2 (all 15 probands analysed) or the 6 additional coding exons (4 probands analysed), thus excluding that, at least for exon 2, mutations other than those at critical residues 24 and 22 may functionally protect CDK4 protein from p16 inhibition.

### Functional analysis of the P48T mutant protein

The p16 protein carrying the novel P48T missense mutation was initially tested for its ability to interact with specific CDKs in a direct in vitro binding assay. The mixed proteins were immunoprecipitated with an antiserum against p16 and analysed by SDS-PAGE. As revealed in Figure 3A, the P48T variant was indistinguishable from wild-type p16 in its ability to interact with CDK4 or CDK6, while the previously documented mutant A20P (Ruas et al, 1999) showed no detectable binding to CDK4 or CDK6. Neither the wild-type nor the variant p16 proteins interacted with CDK2 (data not shown).

In vitro binding to CDK4/6 represents a relatively simple but non-quantitative test of p16 function and previous investigations have revealed a number of cancer-associated p16 variants that retain CDK-binding potential in vitro yet appear functionally impaired in vivo (Ruas et al, 1999). To determine whether the P48T variant belonged to this category, we used a recombinant retrovirus to express this variant in human diploid fibroblasts engineered to express the receptor for ecotropic viruses (TIG-ER cells) (Ruas et al, 1999). By monitoring the proliferation of TIG-ER cells expressing the P48T variant (Figure 3B), we found that this mutant protein had a diminished ability to inhibit cell growth, whereas wild-type p16 caused growth arrest and the empty vector had no effect on cell proliferation.

We additionally assess the function of the P48T variant by an alternative assay based on the ectopic expression of p16 in U2OS cells. These cells were transiently transfected with expression plasmid carrying the P48T mutated allele and analysed by flow cytometry for cell cycle phase redistribution. The DNA histograms (Figure 3C) show that overexpression of wild-type protein led to a specific accumulation of cells in G1 (75.5% versus 45% for control transfectants), as expected. Conversely, overexpression of the mutated p16 protein caused a reduction of G1 phase cells (61%), consistent with a defect in the arrest at the G1/S boundary. The appropriate expression levels of wild-type and mutated p16 protein in transfected cells were verified by protein immunoblotting (Figure 3C).

### DISCUSSION

Mutation analysis of the *CDKN2A* gene in 15 Italian families has revealed a novel germ line variant, the P48T that clearly segregates with melanoma in family 2587, and the missense mutations R24P, G101W (two families) and N71S already known to be disease-associated. The P48T is located in the first ankyrin repeat at a non-conserved amino acid inside the $NH_2$-terminus (AA 9–72) of the protein. As revealed by deletion studies on p16 (Lilischkis et al, 1996), this region is important for the inhibitory activity of p16 like the central domain (AA 73–131), where inactivating missense mutations usually reside. We found that the P48T variant of p16 is functionally impaired in its ability to inhibit the cell growth and cell cycle progression, thus suggesting a causal role for this mutation. However, the in vitro CDK binding assay has shown that the P48T variant retains some activity, placing it in the category of mutants that are partially impaired (Ruas et al, 1999). Interestingly, the P48 residue has been shown to be structurally buried, contributing to the hydrophobic core of the protein, and previous analysis of a different alteration at this residue, P48L, revealed a p16 variant defective in both CDK binding in vitro and cell proliferation inhibition (Ruas et al, 1999). Thus, different alterations of codon 48 have slightly different effects on the function of the protein. The R24P mutation has been reported in one Australian (Holland et al, 1995), one English (Harland et al, 1997) and two French (Soufir et al, 1998) melanoma-prone families. The G101W is a disease-related mutation quite common in melanoma-prone families of European origin (Ghiorzo et al,

© 2001 Cancer Research Campaign

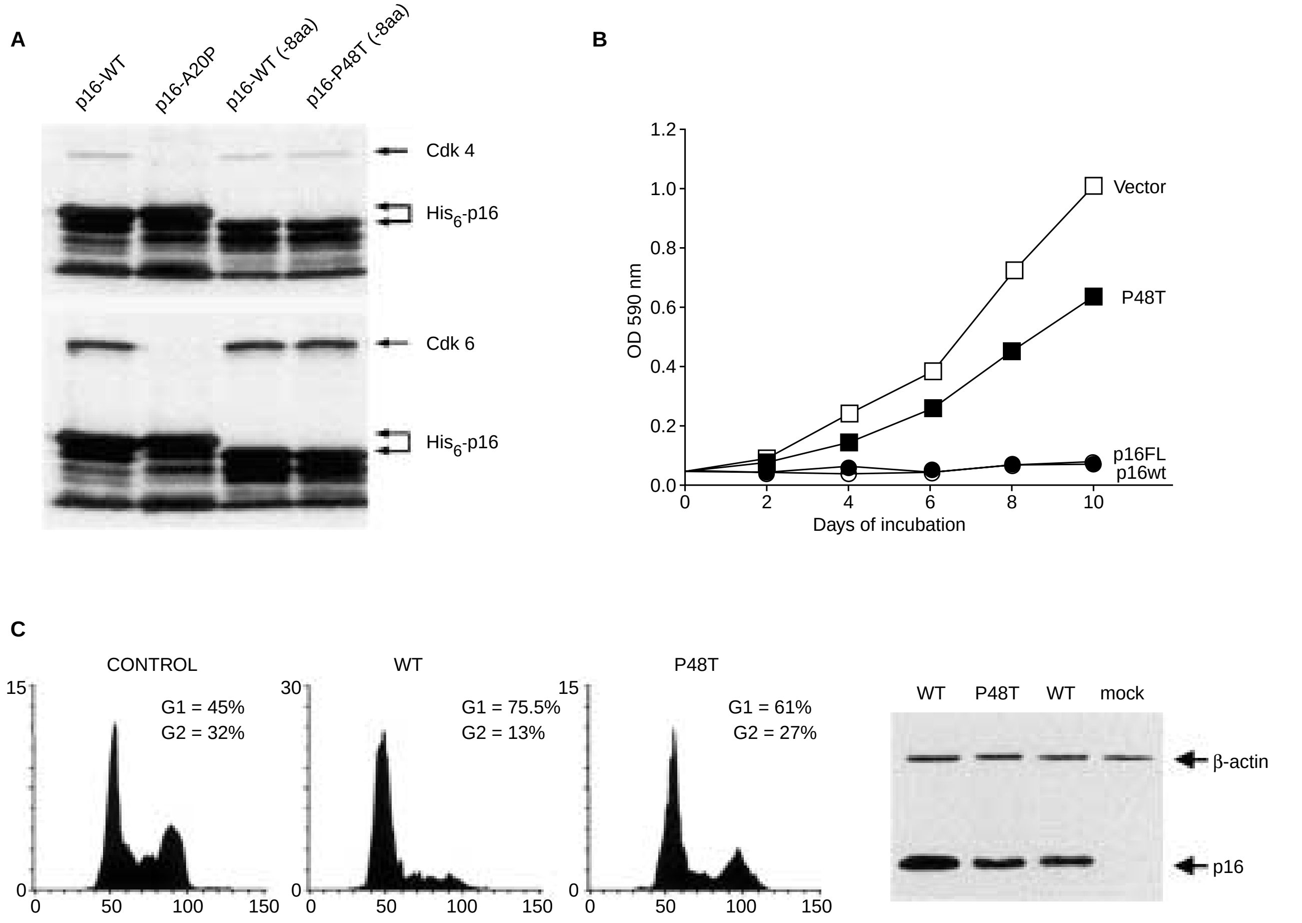

**Figure 3** Functional analyses of the P48T p16 variant. (**A**) Ability of P48T variant to bind CDK4 and CDK6 in vitro. $His_6$-p16 corresponds to full-length (or full-length-8aa) $His_6$-tagged p16; p16 corresponds to a product presumed to initiate at the first methionine in p16 coding region, resulting in a protein without the $His_6$ tag. (**B**) P48T variant is unable to inhibit proliferation of TIG-ER cells. TIG-ER cells were infected with recombinant ecotropic viruses encoding 2HA-tagged p16 proteins and the relative numbers of viable cells were determined by staining with crystal violet and measurement of absorbance at 590 nm. (**C**) P48T variant is unable to inhibit cell cycle progression in U2OS cells. DNA histograms (left side) showing that mutated allele was not as effective as wild-type allele in causing cell cycle arrest after transfection in U2OS cells. Control indicates cells transfected with the CD20 expression plasmid alone. Fluorescence intensity is plotted versus relative cell number. Immunoblot (right side) showing p16 expression levels after transfection with the respective cDNA constructs. Blot was reprobed for β-actin (upper band) to normalize protein loading per lane

1999). Differently, the N71S is only weakly correlated with melanoma risk and regarded by some authors as rare polymorphism, even if absent in normal controls (Ranade et al, 1995). These three variants have all been functionally tested and shown to be partially defective. Notably, the R24P displayed binding activity to CDK6, but not to CDK4 (Harland et al, 1997), and the G101W temperature sensitivity in binding to CDK4 and CDK6 in vitro and in the kinase inhibition and cell cycle arrest assays (Parry and Peters, 1996). The N71S, alike to P48T, retained CDK-binding activity but behaved as partially impaired in the cell proliferation assay (Ruas et al, 1999).

Italian melanoma families commonly have a small number of affected relatives and the likelihood that these families represent clustering of sporadic cases is minor because of the low melanoma incidence in the Italian population (Ghiorzo et al, 1999). In our series of 15 Italian melanoma families, 25% of families with 2 affected relatives and 50% of families with 3 or more affected relatives had identifiable melanoma specific *CDKN2A* mutations. The small sample size of our study may have contributed to these high mutation rates. In other studies on larger clinical populations (Holland et al, 1999; Ruiz et al, 1999; Borg et al, 2000; Goldstein et al, 2000) lower prevalences of mutations have been reported. However, the difference we found in the probability of identifying *CDKN2A* mutations in families of different size is consistent with that reported worldwide and it appears to be dependent on the strength of family history (Soufir et al, 1998). In a recent collaborative study with Mantelli et al (personal communication) on 62 Italian families *CDKN2A* mutations have been found to be significantly more likely in small families when one of the 2 affected members carries multiple primary melanomas or when pancreatic cancer is present in additional family members. This finding can account for the absence of mutations found in most of our families with 2 affected members, as these were free of clinical features predictive of mutation. Additionally, it might imply that some 2-case families of our subset are more likely to represent clustering of sporadic melanomas, or that they carry less penetrant mutant genes. Of note, 2 of the latter families and one family with 3 melanoma cases were characterized by melanoma patients also affected by basal cell carcinoma, thus suggesting a different disease phenotype.

 © 2001 Cancer Research Campaign

In the large family showing segregation of the R24P with melanoma one individual diagnosed with melanoma had no detectable mutated allele at codon 24 in 2 different blood samples, although the age at diagnosis was consistent with that of the other affected relatives. We believe that this individual may be a case of sporadic melanoma (phenocopy), as the segregation of the mutation in 5/6 affected relatives seems unlikely to be a coincidence. Alternatively, co-segregation of another genetic factor cooperating with the *CDKN2A* gene in the predisposition to melanoma could account for the presence of a non-carrier melanoma case and the penetrance of the mutation in this family. The intron 1 variant carried by this individual has not, to our knowledge, been reported before. As this variant, which is not predicted to be critical for protein expression, was not encountered in the other affected or unaffected relatives, we presume that it is a silent variant, possibly of maternal origin, with no role in the development of melanoma.

In our families, evidence for the presence of tumours additional to melanoma indicates a high heterogeneity, as already observed in melanoma families of various geographical origins (Ghiorzo et al, 1999). In studies aimed at investigating whether familial susceptibility to melanoma increases the risk of other tumours, increased risks of pancreatic and breast carcinomas have been found in *CDKN2A* mutation carriers (Goldstein et al, 1995; Borg et al, 2000). Other studies suggest that germ line CDKN2A-impairing function mutations may also predispose to head and neck squamous cell carcinomas in familial melanoma (Yarbrough et al, 1996). The observed number of non-melanoma tumours in our families is too small for a risk analysis. A single case of pancreatic carcinoma was present in our series of families and it occurred in a member of a *CDKN2A* mutation-negative family. Of clinical relevance could be the observation of one P48T mutation carrier from the family 2587 who developed an oral squamous cell carcinoma during the follow-up.

The absence of any *CDK4* mutations in our families supports the conclusion that the activation of this gene is infrequent in familial melanoma and that in this cancer syndrome the deregulation of the RB pathway occurs mainly through mutations in *CDKN2A*. However, we found no *CDKN2A* germ line mutations in a large proportion of families with either 2 or more affected relatives. It is still open to investigation that mutations outside the coding region that result in dysregulation of gene expression might be a possible cause of melanoma predisposition. To date, mutation screening of the *CDKN2A* promoter region in familial melanoma has revealed that causal mutations at this site are rarely present in families with no detectable coding mutations (Harland et al, 2000; Mantelli et al, personal communication).

In conclusion, we confirm in this study the significant role of *CDKN2A* mutations in melanoma-prone families having 3 or more affected relatives. Furthermore, functional data point out that *CDKN2A* mutations may require complementary functional assays to establish their pathogenetic role.

### ACKNOWLEDGEMENTS

We are grateful to Mr Mario Azzini for preparation of the figures, Miss Cristina Mazzadi for secretarial assistance and Mrs Donata Penso for sequencing analysis. This work was supported by grant from AIRC (Associazione Italiana per la Ricerca sul Cancro).

### REFERENCES

Baker SJ, Markowitz S, Fearon ER, Willson JK and Vogelstein B (1990) Suppression of human colorectal carcinoma cell growth by wild-type p53. *Science* **249**: 912–915

Borg A, Sandberg T, Nilsson K, Johannsson O, Klinker M, Masback A, Westerdahl J, Olsson H and Ingvar C (2000) High frequency of multiple melanomas and breast and pancreas carcinomas in CDKN2A mutation-positive melanoma families. *J Natl Cancer Inst* **92**: 1260–1266

Brotherton DH, Dhanaraj V, Wick S, Brizuela L, Domaille PJ, Volyanik, Xu X, Parisini E, Smith BO, Archer SJ, Serrano M, Brenner SL, Blundell TL and Laue ED (1998) Crystal structure of the complex of the cyclin D-dependent kinase Cdk6 bound to the cell-cycle inhibitor p19INK4d. *Nature* **395**: 244–250

Castellano M and Parmiani G (1999) Genes involved in melanoma: an overview of *INK4a* and other loci. *Melanoma Res* **9**: 421–432

Coleman K, Wautlet B, Morrissey D, Mulheron J, Sedman S, Brinkley P, Price S, and Webster K (1997) Identification of CDK4 sequences involved in cyclin D1 and p16 binding. *J Biol Chem* **272**: 18869–18874

Delia D, Goi K, Mizutani S, Yamada T, Aiello A, Fontanella E, Lamorte G, Iwata S, Ishioka C, Krajewski S, Reed CC and Pierotti MA (1997) Dissociation between cell cycle arrest and apoptosis can occur in Li-Fraumeni cells heterozygous for p53 gene mutations. *Oncogene* **14**: 2137–2147

Donghi R, Longoni A, Pilotti S, Michieli P, Della Porta G and Pierotti MA (1993) Gene p53 mutations are restricted to poorly differentiated and undifferentiated carcinomas of the thyroid gland. *J Clin Invest* **91**: 1753–1760

Ghiorzo P, Ciotti P, Mantelli M, Heouaine A, Queirolo P, Rainero ML, Ferrari C, Santi PL, De Marchi R, Farris A, Ajmar F, Bruzzi P and Bianchi-Scarra G (1999) Characterization of ligurian melanoma families and risk of occurrence of other neoplasia. *Int J Cancer* **83**: 441–448

Goldstein AM, Fraser MC, Struewing JP, Hussussian CJ, Ranade K, Zametkin DP, Fontaine LS, Organic SM, Dracopoli NC, Clark WH Jr. et al (1995) Increased risk of pancreatic cancer in melanoma-prone kindreds with p16INK4 mutations. *New Engl J Med* **333**: 970–974

Goldstein AM, Struewing J, Chidambaram A, Fraser MC and Tucker MA (2000) Genotype-phenotype relationships in U.S. melanoma-prone families with CDKN2A and CDK4 mutations. *J Natl Cancer Inst* **92**: 1006–1010

Greene MH and Fraumeni JF, Jr (1979) The hereditary variant of malignant melanoma. In *Human Malignant Melanoma*, Clark WHJr, Goldman LI, Mastangelo MJ (eds) pp 139–166. New York, NY, Grune Stratton

Guldberg P, Kirkin AF, Gronbaek K, thor Straten P, Ahrenkiel V and Zeuthen J (1997) Complete scanning of the CDK4 gene by denaturing gradient gel electrophoresis: a novel missense mutation but low overall frequency of mutations in sporadic metastatic malignant melanoma. *Int J Cancer* **72**: 780–783

Haluska FG and Hodi FS (1998) Molecular genetics of familial cutaneous melanoma. *J Clin Oncol* **16**: 670–682

Harland M, Meloni R, Gruis N, Pinney E, Brookes S, Spurr NK, Frischauf AM, Bataille V, Peters G, Cuzick J, Selby P, Bishop DT and Bishop JN (1997) Germine mutations of the CDKN2 gene in UK melanoma families. *Hum Mol Genet* **6**: 2061–2067

Harland M, Holland EA, Ghiorzo P, Mantelli M, Bianchi-Scarra G, Goldstein AM, Tucker MA, Ponder BAJ, Mann GJ, Bishop DT et al (2000) Mutation screening of the CDKN2A promoter in melanoma families. *Genes Chrom Cancer* **28**: 45–57

Holland EA, Beaton SC, Becker TM, Grulet OM, Peters BA, Rizos H, Kefford RF and Mann GJ (1995) Analysis of the p16 gene, CDKN2, in 17 Australian melanoma kindreds. *Oncogene* **11**: 2289–2294

Holland EA, Schmid H, Kefford RF and Mann GJ (1999) CDKN2A (P16(INK4a)) and CDK4 mutation analysis in 131 Australian melanoma probands: effect of family history and multiple primary melanomas. *Genes Chrom Cancer* **25**: 339–348

Koh J, Enders GH, Dynlacht BD and Harlow E (1995) Tumour-derived p16 alleles encoding proteins defective in cell-cycle inhibition. *Nature* **375**: 506–510

Lilischkis R, Sarcevic B, Kennedy C, Warlters A and Sutherland RL (1996) Cancer-associated mis-sense and deletion mutations impair p16INK4 CDK inhibitory activity. *Int J Cancer* **66**: 249–254

Morgenstern JP and Land H (1990) Advanced mammalian gene transfer: high titre retroviral vectors with multiple drug selection markers and a complementary helper-free packaging cell line. *Nucl Acids Res* **18**: 3587–3596

Parry D and Peters G (1996) Temperature-sensitive mutants of p16CDKN2 associated with familial melanoma. *Mol Cell Biol* **16**: 3844–3852

Ranade K, Hussussian CJ, Sikorski RS, Varmus HE, Goldstein AM, Tucker MA, Serrano M, Hannon GJ, Beach D and Dracopoli NC (1995) Mutations

© 2001 Cancer Research Campaign 

associated with familial melanoma impair p16INK4 function. *Nat Genet* **10**: 114–116
Ruas M and Peters G (1998) The p16INK4a/CDKN2A tumor suppressor and its relatives. *Bioch Biophys Acta* **1378**: F115–F177
Ruas M, Brookes S, McDonald NQ and Gordon P (1999) Functional evaluation of tumor-specific variants of $p16^{INK4a}$/CDKN2A: correlation with protein structure information. *Oncogene* **18**: 5423–5434
Ruiz A, Puig S, Malvehy J, Lazaro C, Lynch M, Gimenez-Arnau AM, Puig L, Sanchez-Conejo J, Estivill X and Castel T (1999) CDKN2A mutations in Spanish cutaneous malignant melanoma families and patients with multiple melanomas and other neoplasia. *J Med Genet* **36**: 490–493
Soufir N, Avril MF, Chompret A, Demenais F, Bpmbled J, Spatz A, Stoppa-Lyonnet D, French Familial Melanoma Study Group, Benard J and Bressac-de Paillerets B (1998) Prevalence of *p16* and *CDk4* germline mutations in 48 melanoma-prone families in France. *Hum Mol Genet* **7**: 209–216
Tsao H, Benoit E, Sober AJ, Thiele C and Haluska FG (1998) Novel mutations in the p16/CDKN2A binding region of the cyclin-dependent kinase-4 gene. *Cancer Res* **58**: 109–113
Wolfel T, Hauer M, Schneider J, Serrano M, Wolfel C, Klehmann-Hieb E, De Plaen E, Hankeln T, Meyer zBK and Beach D (1995) A p16INK4a-insensitive CDK4 mutant targeted by cytolytic T lymphocytes in a human melanoma. *Science* **269**: 1281–1284
Yarbrough WG, Aprelikova O, Pei H, Olshan AF and Liu ET (1996) Familial tumor syndrome associated with a germline nonfunctional $p16^{INK4a}$ allele. *J Natl Cancer Inst* **88**: 1489–1491
Zuo L, Weger J, Yang Q, Goldstein AM, Tucker MA, Walker GJ, Hayward and Dracopoli NC (1996) Germline mutations in the p16INK4a binding domain of CDK4 in familial melanoma. *Nat Genet* **12**: 97–99

 *© 2001 Cancer Research Campaign*
